# Supplementary figures and images for: Optimizing total RNA extraction method for human and mice samples
Source: PeerJ. 2024 Sep 26;12:e18072. doi: 10.7717/peerj.18072 (PMC11439393; doi:10.7717/peerj.18072)

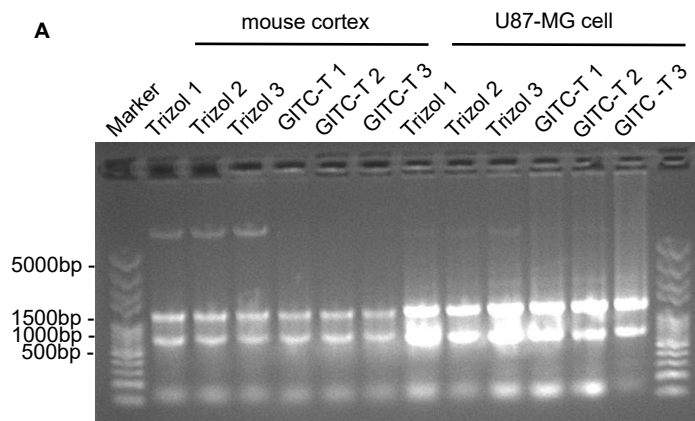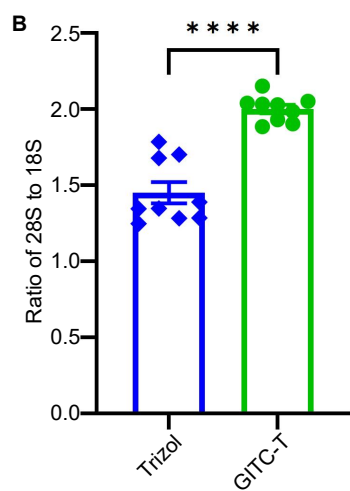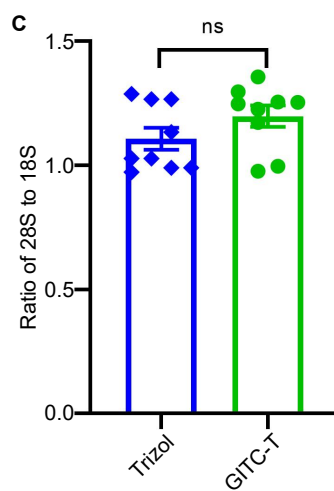

Supplement: Supplemental Information 1 — (A) RNA electrophoresis patterns of different samples extracted by the Trizol and GITC-T methods. (B) The intensity ratio of 28S to 18S in (A) was statistically compared to reflect the difference in the integrity of RNA extracted by the GITC-T and Trizol methods. [file peerj-12-18072-s001.pdf]

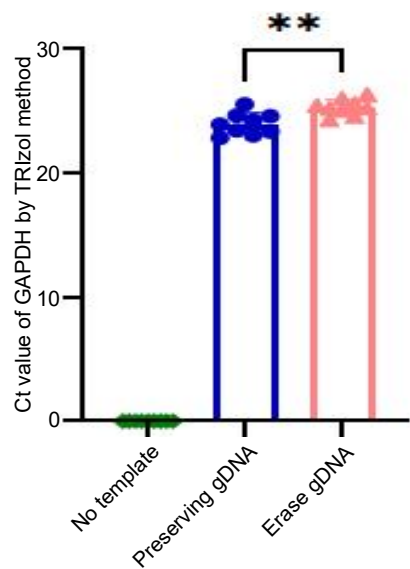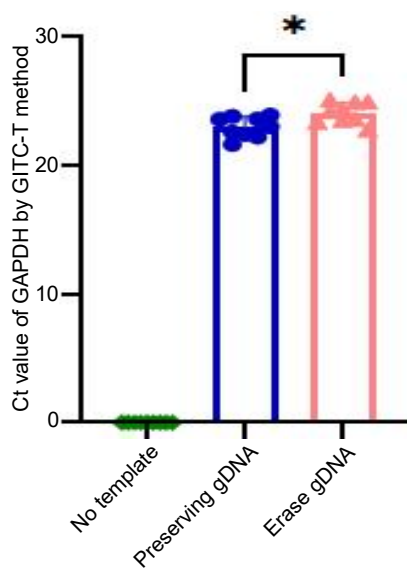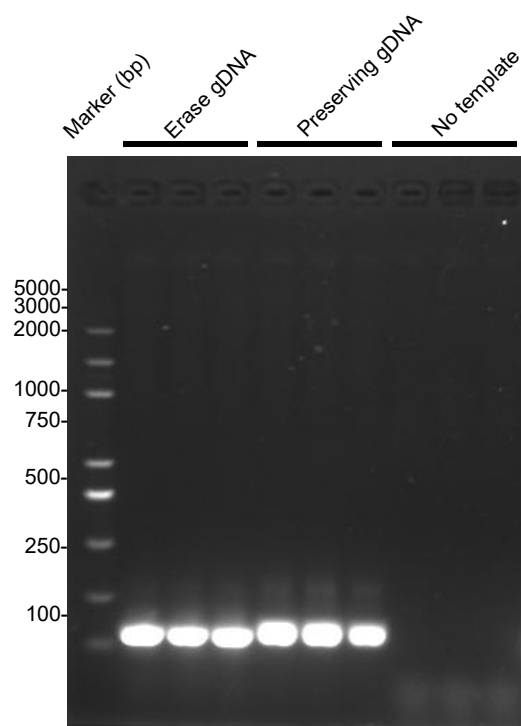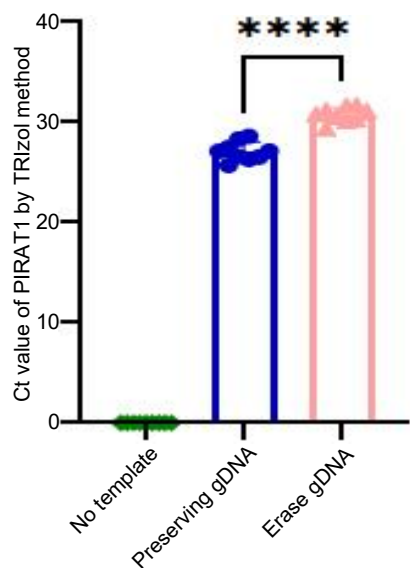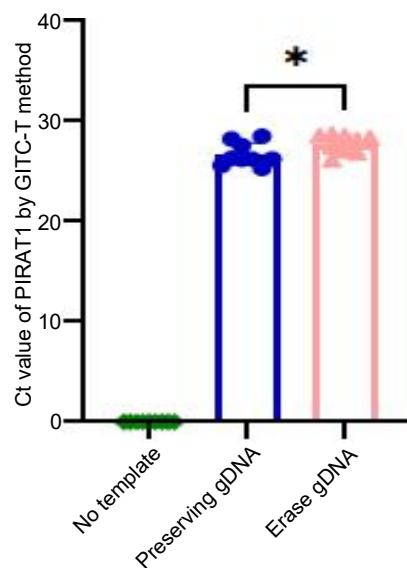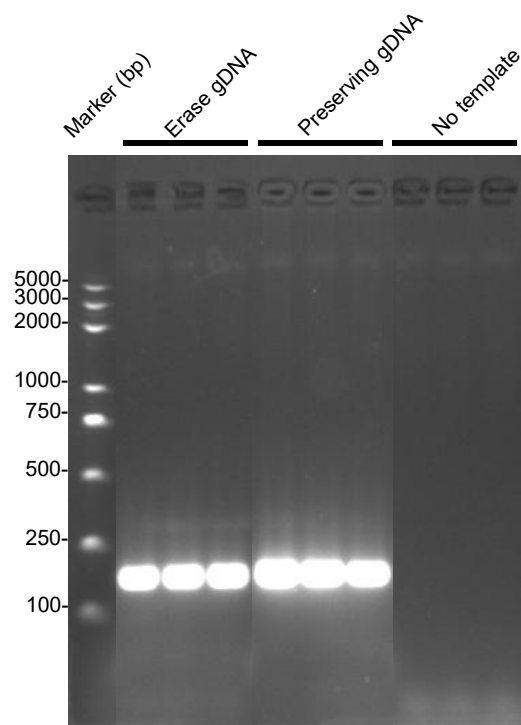

Supplement: Supplemental Information 2 — Based on the q-PCR results from Fig. 5B, statistical analysis of the three sample groups shows that gDNA residues are present in the total RNA extracted by both methods. However, the GITC-T method has relatively less residual gDNA, resulting in little impact on the GAPDH (upper panel) and PIRAT1 (lower panel) genes Ct values between the groups with and without gDNA removal. On the far right is the electrophoresis image of the q-PCR products for the GAPDH and PIRAT1 genes, which visually illustrates the impact of residual gDNA in the total RNA samples. [file peerj-12-18072-s002.pdf]

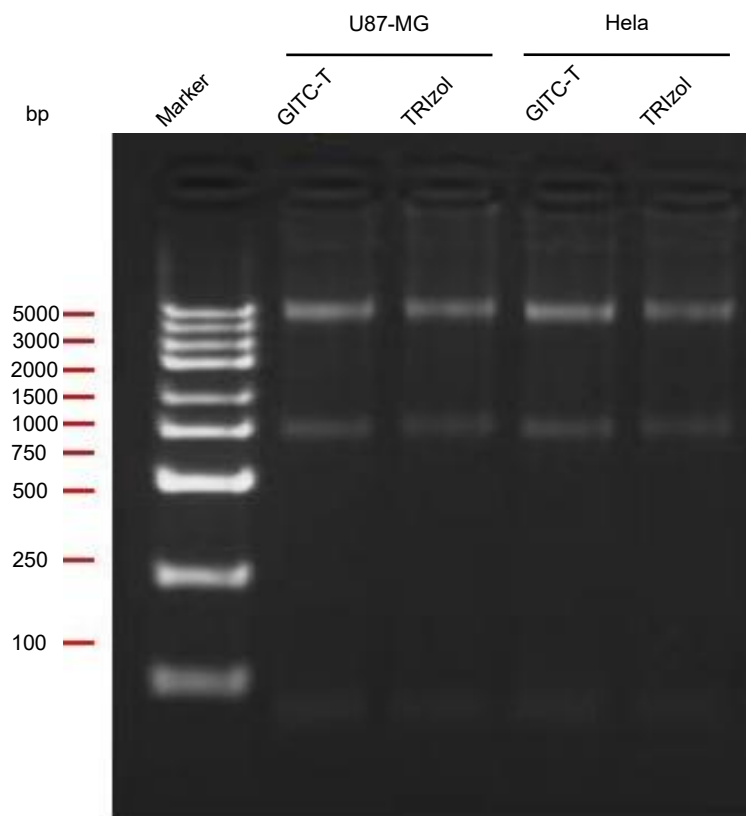

Supplement: Supplemental Information 3 [file peerj-12-18072-s003.pdf]
