# Supplementary material for: Optimizing total RNA extraction method for human and mice samples
Source: PeerJ. 2024 Sep 26;12:e18072. doi: 10.7717/peerj.18072 (PMC11439393; doi:10.7717/peerj.18072)
Supplement: Supplemental Information 4 [file peerj-12-18072-s004.docx]

|  | | |
| --- | --- | --- |
| **Reagent** | **source** | **identifier** |
| TRIzol | Invitrogen | Cat# 15596026 |
| SDS | Biosharp | Cat# 21089245 |
| GITC | Macklin | Cat# 14606154 |
| Trichloromethane | Chron Chemicals | Cat# 2019081601 |
| Isopropanol | Chron Chemicals | Cat# 2022041802 |
| 75% ethanol | Sichuan JinShan Pharmaceutical Co,Ltd | Cat# 211201 |
| Tris | BioFroxx | Cat# 1115GR500 |
| agarose | Biowest | Cat# 111860 |
| GAPDH primer | Sangon Biotech | Cat# 2314252636 |
| PIRAT1 primer | Sangon Biotech | Cat# 2314262638 |
| BME | Gibco | Cat# 21010046 |
| F-12K | Gibco | Cat# 21127030 |
| FBS | Gibco | Cat# 12484028 |
| Trypsin-EDTA Solution | Biosharp | Cat# BL501A |
| Penicillin-Streptomycin Solution | Biosharp | Cat# BL505A |
| DEPC water | Biosharp | Cat# BL510A |
| DPBS | Biosharp | Cat# BL310A |
| R-T Kit | TaKaRa | Cat# RR047A |
| q-PCR Kit | TaKaRa | Cat# RR820A |
