# Supplementary material for: Optimizing total RNA extraction method for human and mice samples
Source: PeerJ. 2024 Sep 26;12:e18072. doi: 10.7717/peerj.18072 (PMC11439393; doi:10.7717/peerj.18072)
Supplement: Supplemental Information 5 [file peerj-12-18072-s005.docx]

|  | |
| --- | --- |
| **instruments** | **source** |
| Homogenization | Tengen Biochemical Technology |
| NanoDrop™ One Micro-volume UV-Vis Spectrophotometer | Thermo Fisher Scientific |
| Mini Ready Sub-Cell GT Horizontal Electrophoresis System | Bio-Rad Laboratories |
| Gel documentation imaging system | Clinx Science Instruments Co |
| CFX Opus 96 Real-Time PCR System | Bio-Rad Laboratories |
| T100TM Thermal Cycler | Bio-Rad Laboratories |
| Steri-Cycle i160 CO2 Incubator | Thermo Fisher scientific |
| Scientific Biological Safety Cabinets | Thermo Fisher scientific |
