# Supplementary material for: Optimizing total RNA extraction method for human and mice samples
Source: PeerJ. 2024 Sep 26;12:e18072. doi: 10.7717/peerj.18072 (PMC11439393; doi:10.7717/peerj.18072)
Supplement: Supplemental Information 6 [file peerj-12-18072-s006.docx]

|  | |
| --- | --- |
| **Gene** | **Sequence of primer** |
| Human-132-GAPDH | Forward: CTTTGGTATCGTGGAAGGACTC |
|  | Reverse: GTAGAGGCAGGGATGATGTTCT |
| Human-159-PIRAT1 | Forward: AACGACAGCTTGCAAAGCAC |
|  | Reverse: GCCAAAGGCCAGACAAACAA |
