# Supplementary material for: Optimizing total RNA extraction method for human and mice samples
Source: PeerJ. 2024 Sep 26;12:e18072. doi: 10.7717/peerj.18072 (PMC11439393; doi:10.7717/peerj.18072)
Supplement: Supplemental Information 7 [file peerj-12-18072-s007.docx]

|  | |
| --- | --- |
| **Component** | **Dosage** (μl) |
| Rnase-free water | 9.5 |
| 2×TB Green Premix Ex Taq Ⅱ Fast qPCR | 12.5 |
| 10 μM Forward Primer | 1 |
| 10 μM Reverse Primer | 1 |
| Template cDNA | 1 (≈17 ng ) |
| Total | 25 |
