# Supplementary material for: Optimizing total RNA extraction method for human and mice samples
Source: PeerJ. 2024 Sep 26;12:e18072. doi: 10.7717/peerj.18072 (PMC11439393; doi:10.7717/peerj.18072)
Supplement: Supplemental Information 8 [file peerj-12-18072-s008.docx]

|  | | | | |
| --- | --- | --- | --- | --- |
| **sample** | **protocol** | **RNA concentration**(ng/ul) | **OD**_260/280_ | **OD**_260/230_ |
| U87-MG | TRIzol | 907.13±204.70 | 1.97±0.019 | 1.83±0.165 |
|  | GITC-T | 1065.27±22.17 | 1.98±0.005 | 2.02±0.024 |
| Hela S3 | TRIzol | 931.33±53.57 | 1.96±0.022 | 1.99±0.025 |
|  | GITC-T | 1049±68.65 | 1.99±0.012 | 2.02±0.082 |
| blood | TRIzol | 245.7±63.57 | 1.78±0.038 | 0.99±0.178 |
|  | GITC-T | 290.53±55.37 | 1.83±0.038 | 1.06±0.135 |
| serum | TRIzol | 25.4±9.87 | 1.4±0.014 | 0.28±0.009 |
|  | GITC-T | 44±24.43 | 1.41±0.006 | 0.24±0.041 |
| plasma | TRIzol | 30.47±16.61 | 1.44±0.032 | 0.30±0.005 |
|  | GITC-T | 38.13±6.67 | 1.56±0.061 | 0.29±0.148 |
